# Two-Dimensional Augmented State–Space Approach with Applications to Sparse Representation of Radar Signatures

**DOI:** 10.3390/s19214631

**Published:** 2019-10-24

**Authors:** Kejiang Wu, Xiaojian Xu

**Affiliations:** School of Electronics and Information Engineering, Beihang University, Beijing 100191, China; wukejiang88@buaa.edu.cn

**Keywords:** damped exponential (DE) model, inverse synthetic aperture radar (ISAR), radar signatures, state–space approach (SSA), sparse representation

## Abstract

In this work, we focus on sparse representation of two-dimensional (2-D) radar signatures for man-made targets. Based on the damped exponential (DE) model, a 2-D augmented state–space approach (ASSA) is proposed to estimate the parameters of scattering centers on complex man-made targets, i.e., the complex amplitudes and the poles in down-range and aspect dimensions. An augmented state–space approach is developed for pole estimation of down-range dimension. Multiple-range search strategy, which applies one-dimensional (1-D) state–space approach (SSA) to the 1-D data for each down-range cell, is used to alleviate the pole-pairing problem occurring in previous algorithms. Effectiveness of the proposed approach is verified by the numerical and measured inverse synthetic aperture radar (ISAR) data.

## 1. Introduction

Sparse representation of two-dimensional (2-D) radar signatures has been widely used in many applications, such as super-resolution radar imaging, data compression, and target identification [1,2,3,4]. 2-D radar signatures can be reconstructed with fewer data, where a set of parameters including the locations, amplitudes, and damping factors are used to represent the returned signals from spatial distributed scattering centers. Moreover, 2-D ultra-wideband images of radar targets can be obtained through parameter interpolation or extrapolation [5].

Over the years, several model-based spectral estimation approaches have been developed for sparse representation of 2-D radar signatures, in which the common idea of these approaches is the development of a parametric model based on electromagnetic scattering mechanisms. In this way, a set of parameters may be found to represent the original signatures. Please note that most sparse representation approaches are considered to have super-resolution capabilities because they are supposed to estimate the parameters of scattering centers that cannot be distinguished by standard processing [6,7,8,9,10,11,12,13,14,15,16,17,18,19,20,21]. Typically, these approaches include the amplitude and phase estimation of a sinusoid (APES) algorithm [6], fast Fourier transform (FFT)-based technique (CLEAN) [7,8], the compressed sensing (CS)-based procedure [9,10,11] and the subspace-based approach [12,13,14,15,16,17,18,19,20,21]. The APES algorithm could provide accurate estimation of the complex amplitudes whereas it does not estimate the locations of scattering centers. The 2-D CLEAN technique is a computationally efficient procedure which uses the undamped exponential model and deconvolution algorithm for optimization [7,22]. The main process of the CS-based procedure is to represent the signatures using an over-complete dictionary and the corresponding coefficients [9,10]. The performance of this approach depends on the initial coefficients and the threshold used in terminating iterations.

Another important group of 2-D sparse representation technique is the subspace-based approaches. The representative algorithms of this kind include the 2-D multiple signal classification (MUSIC) [12], matrix enhancement and matrix pencil (MEMP) [13,14], 2-D total least squares (TLS) Prony [15], algebraically coupled matrix pencils (ACMP) [16], 2-D estimation of signal parameters via rotational invariance techniques (2-D ESPRIT) [17] and 2-D system realization technique [18,19]. The rationale and pole-pairing scheme of these algorithms are listed in Table 1.

While these subspace-based algorithms are demonstrated to be useful for the signatures of targets or simulated scattering centers, there are still some technique challenges, especially for sparse representation of the wideband radar signatures collected on complex man-made targets. First, the pairing schemes used by many existing algorithms cannot provide correct pole pairs in certain circumstances. i.e., MEMP, ACMP and 2-D system realization meet with the pole-pairing problem when there are numerous repeated poles in either the down-range or aspect dimension [17,20,23]. Second, the model order, which directly affects the result of parameter estimation such as 2-D MUSIC, MEMP, and 2-D ESPRIT et al., is difficult to be determined in the measurement environment [8]. Third, the compromises between the computational complexity and accuracy should be considered in the application of these algorithms [12]; for instance, the 2-D TLS Prony has smaller computational complexity than MEMP but with loss in accuracy [15].

In this paper, we focus on developing an approach for sparse representation of 2-D radar signatures collected on man-made targets. An augmented state–space approach is proposed for pole estimation of the down-range dimension. Multiple-range search strategy is then applied to estimate the pairing poles and corresponding amplitudes along the aspect dimension. Compared to the existing methods [14,17,18], the advantages of the 2-D augmented state–space approach (ASSA) are: (1) Computational complexity of the algorithm is much reduced since the newly defined Hankel matrix and several time-saving operations are adopted, whereas the pole-estimation accuracy is still at the same level; (2) The pole-pairing problem can be alleviated because all the poles are adaptively paired by using the multiple-range search strategy; And (3) an eigenvalue sequences transform algorithm is proposed, which could provide fast model order selection.

The remainder of the paper is organized as follows. Section 2 briefly presents the damped exponential models for 1-D and 2-D signals. In Section 3, a two-step procedure for 2-D ASSA is developed. Results for numerical and measured inverse synthetic aperture radar (ISAR) data representation are demonstrated to validate the effectiveness of the proposed procedure in Section 4. We conclude the paper in Section 5. Appendices are given to show more mathematical details of the proposed approach.

## 2. Data Model

### 2.1. 1-D Damped Exponential Model

As described in DE model [22,24], the 1-D radar signatures $y\left(f_{n}\right)$ can be expressed as a summation of *K* scattering centers corrupted with noise w(n).

(1) $$\begin{array}{lll} y\left(f_{n}\right) & = & \sum_{k=1}^{K}A_{k}\exp\left[\left(\beta_{k}+j2\pi \frac{r_{k}}{c}\right)f_{n}\right]+w\left(n\right)\\ & = & \sum_{k=1}^{K}a_{k}p_{k}^{n}+w\left(n\right) \end{array}$$

where *n* = 1,2,…,*N* and *N* denotes the number of pulses, *A_k_* is the complex amplitude of the *k*-*th* scattering center; *β_k_* is the damping factor with respect to frequency; $r_{k}$ denotes the relative range; The parameter *f_n_* denotes the radar frequency $f_{n}=f_{c}+\left(n-1-N2\right)\Delta f$ where $f_{c}$ is the center frequency and *N*2 = *ceil*(*N*/2) denotes the smallest integer less than or equal to *N*/2; $a_{k}$ represents the amplitude of the *k*-*th* scattering center in pole form, the pole $p_{k}=\exp\left[\left(\beta_{k}+j2\pi r_{k}/c\right)\Delta f\right]$ represents the transfer function of the *k*-*th* scattering center; *c* = 3 × 10^8^ m/s is the propagation velocity. It worth noting that the data models used in this paper are all considered in a stepped frequency radar [25,26].

According to the discrete-time control theory and auto-regressive moving average (ARMA) model, Piou and Naishadham proposed a one-dimensional state–space approach (1-D SSA) which use a state–space description to the 1-D radar signatures in (1) [22].

(2) $$x\left(n+1\right)=Ax\left(n\right)+Bu\left(n\right)$$

(3) $$y\left(n\right)=Cx\left(n\right)+u\left(n\right)$$

where $x\left(n\right)\in {\mathbb{C}}^{K\times 1}$ is the state vector, $y\left(n\right)$ denotes the signal sequence $y\left(f_{n}\right)$, $u\left(n\right)$ is the input vector, $A\in {\mathbb{C}}^{K\times K}$ represents the open-loop matrix, $B\in {\mathbb{C}}^{K\times 1}$ and $C\in {\mathbb{C}}^{1\times K}$ are the constant matrices. Thus, the 1-D noiseless radar signatures same as (1) can be expressed as

(4) $$\left[\begin{array}{cccc} \tilde{y}(1) & \tilde{y}(2) & \ldots & \tilde{y}(N) \end{array}\right]=\left[\begin{array}{cccc} CB & CAB & \ldots & CA^{N-1}B \end{array}\right]$$

As described in [22], 1-D SSA could precisely estimate the state matrices **A**, **B**, and **C**. Once these three state matrices are computed, the model parameters in (1) can be estimated by using the eigen-decomposition technique.

### 2.2. 2-D Damped Exponential Model

As a 2-D extension of 1-D DE model, the radar signatures obtained from different aspect angles are considered to be the summation of a finite number of dispersive scattering centers [13,14,15,16,17,18]. Typically, it is applicable to modeling the 2-D radar signatures with small aspect ranges [27].

(5) $$\begin{array}{lll} y(\theta_{m},f_{n}) & = & \sum_{k=1}^{K}A_{k}\exp\left[\left(\frac{j2\pi r_{1k}}{c}+\beta_{1k}\right)f_{c}\theta_{m}\right]\exp\left[\left(\frac{j2\pi r_{2k}}{c}+\beta_{2k}\right)f_{n}\right]+w(m,n)\\ & = & \sum_{k=1}^{K}a_{k}s_{k}^{m}p_{k}^{n}+w(m,n) \end{array}$$

in matrix notation, the 2-D radar signatures in (5) can be expressed as:

(6) $$Y=\left[\begin{array}{cccc} y(1,1) & y(1,2) & \ldots & y(1,N)\\ y(2,1) & y(2,2) & \ldots & y(2,N)\\ \vdots & \vdots & \ldots & \vdots \\ y(M,1) & y(M,2) & \ldots & y(M,N) \end{array}\right]$$

where m=1,…,M and n=1,…,N; {$r_{1k}$,$r_{2k}$} give the relative locations of the *k*-*th* scattering center; {$\beta_{1k}$, $\beta_{2k}$} characterize the frequency and aspect dependence of scattering; $\theta_{m}=\theta_{0}+(m-1)\Delta \theta$ denotes the *m*-*th* aspect angle where $\theta_{0}$ is the starting angle and Δθ represents the angle interval; w(m,n) is the Gaussian noise with zero-mean; y(m,n) denotes the signal sequences $y\left(\theta_{m},f_{n}\right)$; {$s_{k}$, $p_{k}$} refer to poles of the down-range and aspect dimension.

(7) $$s_{k}=\exp\left[\left(\frac{j2\pi r_{1k}}{c}+\beta_{1k}\right)f_{c}\Delta \theta \right]$$

(8) $$p_{k}=\exp\left[\left(\frac{j2\pi r_{2k}}{c}+\beta_{2k}\right)\Delta f\right]$$

## 3. Two-Dimensional Augmented State–Space Approach

From the 2-D DE model in (5), the vector $\tilde{Y}(n)$, which represents the *n*-th column of the noiseless signature matrix $\tilde{Y}=Y-W$ (where W is the noise matrix), can be decomposed as.

(9) $$\tilde{Y}(n)=\left[\begin{array}{cccc} a_{1}s_{1}^{1}p_{1} & a_{2}s_{2}^{1}p_{2} & \vdots & a_{K}s_{K}^{1}p_{K}\\ a_{1}s_{1}^{2}p_{1} & a_{2}s_{2}^{2}p_{2} & \vdots & a_{K}s_{K}^{2}p_{K}\\ \vdots & \vdots & \vdots & \vdots \\ a_{1}s_{1}^{M}p_{1} & a_{2}s_{2}^{M}p_{2} & \vdots & a_{K}s_{K}^{M}p_{K} \end{array}\right]{\left[\begin{array}{cccc} p_{1} & 0 & \vdots & 0\\ 0 & p_{2} & \vdots & 0\\ \vdots & \vdots & \vdots & \vdots \\ 0 & 0 & \vdots & p_{K} \end{array}\right]}^{n-1}l_{K}$$

where K denotes the number of scattering centers, $l_{K}$ indicates the column vector of ones with length *K*.

Actually, we often meet the one-to-multiple matching situation, i.e., one pole $p_{k}$ is corresponding to more than one poles $s_{k1},s_{k2},\ldots$. These repeated poles can be merged and (9) could be rewritten as.

(10) $$\tilde{Y}(n)=\left[\begin{array}{cccc} \sum_{t=1}^{l_{1}}a_{1t}s_{1t}p_{1} & \sum_{t=1}^{l_{2}}a_{2t}s_{2t}p_{2} & \ldots & \sum_{t=1}^{l_{K_{1}}}a_{K_{1}t}s_{K_{1}t}p_{K_{1}}\\ \sum_{t=1}^{l_{1}}a_{1t}s_{1t}^{2}p_{1} & \sum_{t=1}^{l_{2}}a_{2t}s_{2t}^{2}p_{2} & \ldots & \sum_{t=1}^{l_{K_{1}}}a_{K_{1}t}s_{K_{1}t}^{2}p_{K_{1}}\\ \vdots & \vdots & \vdots & \vdots \\ \sum_{t=1}^{l_{1}}a_{1t}s_{1t}^{M}p_{1} & \sum_{t=1}^{l_{2}}a_{2t}s_{2t}^{M}p_{2} & \vdots & \sum_{t=1}^{l_{K_{1}}}a_{K_{1}t}s_{K_{1}t}^{M}p_{K_{1}} \end{array}\right]{\left[\begin{array}{cccc} p_{1} & 0 & \vdots & 0\\ 0 & p_{2} & \vdots & 0\\ \vdots & \vdots & \vdots & \vdots \\ 0 & 0 & \vdots & p_{K} \end{array}\right]}^{n-1}l_{K_{1}}$$

where $K_{1}$ represents the number of non-repeated poles in the down-range dimension. $l_{1},l_{2},\ldots l_{K_{1}}$ denote the number of repeated poles, respectively, for the corresponding poles $p_{1},p_{2},\ldots p_{K_{1}}$.

We define the matrix **P** as.

(11) $$P=Q\left[\begin{array}{cccc} p_{1} & 0 & \vdots & 0\\ 0 & p_{2} & \vdots & 0\\ \vdots & \vdots & \vdots & \vdots \\ 0 & 0 & \vdots & p_{K_{1}} \end{array}\right]Q^{*}$$

where * denotes the Hermitian operator; **Q** is a unitary matrix which satisfies $Q^{*}Q=QQ^{*}=E$, **E** is the identity matrix.

Two constant matrices, i.e., **S** and **D**, are defined to simplify the expression in (10).

(12) $$S=\left[\begin{array}{cccc} \sum_{t=1}^{l_{1}}a_{1t}s_{1t}p_{1} & \sum_{t=1}^{l_{2}}a_{2t}s_{2t}p_{2} & \ldots & \sum_{t=1}^{l_{K_{1}}}a_{K_{1}t}s_{K_{1}t}p_{K_{1}}\\ \sum_{t=1}^{l_{1}}a_{1t}s_{1t}^{2}p_{1} & \sum_{t=1}^{l_{2}}a_{2t}s_{2t}^{2}p_{2} & \ldots & \sum_{t=1}^{l_{K_{1}}}a_{K_{1}t}s_{K_{1}t}^{2}p_{K_{1}}\\ \vdots & \vdots & \vdots & \vdots \\ \sum_{t=1}^{l_{1}}a_{1t}s_{1t}^{M}p_{1} & \sum_{t=1}^{l_{2}}a_{2t}s_{2t}^{M}p_{2} & \vdots & \sum_{t=1}^{l_{K_{1}}}a_{K_{1}t}s_{K_{1}t}^{M}p_{K_{1}} \end{array}\right]Q*$$

(13) $$D=Ql_{K_{1}}$$

Using (11)–(13), expression in (10) is simplified as.

(14) $$\tilde{Y}(n)=SP^{n-1}D$$

Thus, the noiseless signatures $\tilde{Y}$ can be written as.

(15) $$\tilde{Y}=\left[\begin{array}{cccc} SD & SPD & \ldots & SP^{N-1}D \end{array}\right]$$

Comparing (15) with (4), it can be found that these two equations have extremely similar structures. Thus, the parameters $a_{k}$, $s_{k}$ and $p_{k}$ can be estimated by 2-D augmentation of 1-D SSA, which is called 2-D augmentation state–space approach (2-D ASSA).

Here, the 2-D ASSA consists of two steps. An augmented state–space approach is applied to estimating the pole $p_{k}$ in down-range dimension. Then multiple-range search strategy is used for estimating the matching pole $s_{k}$ as well as the corresponding amplitude $a_{k}$. Details are as follows.

### 3.1. Pole Estimation of the Down-Range Dimension

First, we introduce a single augmented Hankel matrix H of size M(N−L+1)×L by analogy of the enhanced Hankel matrix in 1-D SSA:

(16) $$H=\left[\begin{array}{cccc} Y\left(1\right) & Y\left(2\right) & \ldots & Y\left(L\right)\\ Y\left(2\right) & Y\left(3\right) & \ldots & Y\left(L+1\right)\\ \vdots & \vdots & \ldots & \vdots \\ Y\left(N-L+1\right) & Y\left(N-L+2\right) & \ldots & Y\left(N\right) \end{array}\right]$$

where each element Y(n), first mentioned in (9), represents the *n*-th column of the matrix **Y**; *L* is the step size of the correlation window, which is heuristically set to be *L*=N2 [22].

Next, do the singular value decomposition (SVD) of the Hankel matrix.

(17) $$H=[\begin{array}{cc} U_{sn} & U_{n} \end{array}]\left[\begin{array}{cc} {ℜ}_{sn} & \\ & {ℜ}_{n} \end{array}\right]\left[\begin{array}{c} V_{sn}^{*}\\ V_{n}^{*} \end{array}\right]$$

where $U_{sn}$,$U_{n}$,$V_{sn}^{*}$ and $V_{n}^{*}$ are unitary matrices; ${ℜ}_{sn}$ and ${ℜ}_{n}$ are diagonal matrices; The matrices with subscript ‘*sn*’ refer to the components of signal space and those matrices with subscript ‘*n*’ denote the components of noise space; The rank of the noiseless matrix $U_{sn}{ℜ}_{sn}V_{sn}^{*}$ is known as the model order which has been widely used in [14,22,27].

As proved in [14,17], the model order in the down-range dimension is equal to $K_{1}$ and should be less than the number of columns or rows of **H** at least. Thus, the number of non-repeated poles $K_{1}$ should satisfy the following condition.

(18) $$\left\{\begin{array}{c} MN-ML+M\geq K_{1}\\ L\geq K_{1} \end{array}\right.$$

Model order selection is an inevitable problem in modeling the 2-D signatures [14,22,27]. A series of eigenvalue-based criteria, such as Akaike information criterion (AIC), minimum description length (MDL) criterion and the minimum eigenvalue (MEV) criterion, have been proposed for solving this problem [28,29]. Those criteria are useful in model order selection but with time consuming process [30]. Here an eigenvalue sequences transform algorithm is proposed for estimating the model order due to its low computational complexity. This algorithm can provide fast estimation but with loss in robustness to noise. More details of this algorithm are presented in Appendix A.

Based on the linear systems theory [31] and the matrix expression in (14) and (16), the noiseless matrix $\tilde{H}$ can be further factorized as:

(19) $$\tilde{H}=U_{sn}{ℜ}_{sn}V_{sn}^{*}=\Omega \Gamma$$

where Ω is the $(N-L+1)M\times K_{1}$ observability matrix which can be further expressed by the matrices **S** and **P**.

(20) $$\Omega =U_{sn}{ℜ}_{sn}^{1/2}={\left[\begin{array}{cccc} S & SP & \ldots & SP^{N-L} \end{array}\right]}^{T}$$

and Γ is the $K_{1}\times L$ controllability matrix which can be expressed by the matrices **P** and **D**.

(21) $$\Gamma ={ℜ}_{sn}^{1/2}V_{sn}^{*}=\left[\begin{array}{cccc} D & PD & \ldots & P^{L-1}D \end{array}\right]$$

Considering that computational complexity of (19)–(21) is enlarged observably for large data sets, operations (22)–(24) are used for alternative steps which have lower computational load but with minimal calculation error.

(22) $${\tilde{H}}^{*}\tilde{H}=V_{sn}{ℜ}_{sn}^{2}V_{sn}^{*}=V_{sn}ZV_{sn}^{*}$$

(23) $$\Omega \approx HV_{sn}Z^{-1/4}$$

(24) $$\Gamma =Z^{-1/4}V_{sn}^{*}$$

where $Z={ℜ}_{sn}^{2}$.

As an analogy of the open-loop matrix in 1-D SSA, the augmented open-loop matrix **P** can be derived from the observability matrix by using least square.

(25) $$P={\left(\Omega_{rf}^{*}\Omega_{rf}\right)}^{-1}\Omega_{rf}^{*}\Omega_{rl}$$

Or it can be computed by the controllability matrix Γ.

(26) $$P=\Gamma_{cl}\Gamma_{cf}^{*}{\left(\Gamma_{cf}\Gamma_{cf}^{*}\right)}^{-1}$$

where $\Omega_{rf}$ is the first (*N* − *L*)*M* rows of Ω and $\Omega_{rl}$ denotes the last (*N* − *L*)*M* rows of Ω; $\Gamma_{cf}$ represents the first (*L* − 1) columns of Γ and $\Gamma_{cl}$ is the last (*L* − 1) columns of Γ. From (20) and (21), these matrices can be rewritten as

(27) $${\left[\begin{array}{cccc} S & SP & \ldots & SP^{N-L-1} \end{array}\right]}^{T}=\Omega_{rf}$$

(28) $${\left[\begin{array}{cccc} SP & SP^{2} & \ldots & SP^{N-L} \end{array}\right]}^{T}=\Omega_{rl}$$

(29) $$\left[\begin{array}{cccc} D & PD & \ldots & P^{L-2}D \end{array}\right]=\Gamma_{cf}$$

(30) $$\left[\begin{array}{cccc} PD & P^{2}D & \ldots & P^{L-1}D \end{array}\right]=\Gamma_{cl}$$

More details of the derivation of the matrices **S**, **P**, and **D** are listed in Appendix B.

According to (11), the vector $\left[\begin{array}{cccc} p_{1} & p_{2} & \ldots & p_{K_{1}} \end{array}\right]$ is obtained by performing the SVD.

(31) $$\Lambda =Q^{*}PQ$$

(32) $$diag(\Lambda )=\left[\begin{array}{cccc} p_{1} & p_{2} & \ldots & p_{K_{1}} \end{array}\right]$$

where diag(Λ) denotes the diagonal element of matrix Λ.

### 3.2. Pole Estimation of the Aspect Dimension

In this step, multiple-range search, which applies one-dimensional (1-D) state–space approach (SSA) to the 1-D data for each down-range cell, is used for pole estimation of the aspect dimension and pole adaptive pairing. Details are as follows.

We introduce the Vandermonde sub-matrix **O**.

(33) $$O=\left[\begin{array}{cccc} p_{1}^{1} & p_{1}^{2} & \vdots & p_{1}^{N}\\ p_{2}^{1} & p_{2}^{2} & \vdots & p_{2}^{N}\\ \vdots & \vdots & \vdots & \vdots \\ p_{K_{1}}^{1} & p_{K_{1}}^{2} & \vdots & p_{K_{1}}^{N} \end{array}\right]$$

From (5), the noiseless signatures $\tilde{Y}$ can be factorized as a product of the pairing matrix G and **O**, where each column of G is associated with each row of **O**.

(34) $$\tilde{Y}=\left[\begin{array}{cccc} \sum_{t=1}^{l_{1}}a_{1t}s_{1t} & \sum_{t=1}^{l_{2}}a_{2t}s_{2t} & \ldots & \sum_{t=1}^{l_{K_{1}}}a_{K_{1}t}s_{K_{1}t}\\ \sum_{t=1}^{l_{1}}a_{1t}s_{1t}^{2} & \sum_{t=1}^{l_{2}}a_{2t}s_{2t}^{2} & \ldots & \sum_{t=1}^{l_{K_{1}}}a_{K_{1}t}s_{K_{1}t}^{2}\\ \vdots & \vdots & \vdots & \vdots \\ \sum_{t=1}^{l_{1}}a_{1t}s_{1t}^{M} & \sum_{t=1}^{l_{2}}a_{2t}s_{2t}^{M} & \vdots & \sum_{t=1}^{l_{K_{1}}}a_{K_{1}t}s_{K_{1}t}^{M} \end{array}\right]O=GO$$

Thus, the pairing matrix G in (34) can be calculated by using least square, i.e.

(35) $$G=\tilde{Y}O^{*}{\left(OO^{*}\right)}^{-1}$$

The *k*-th column of the pairing matrix G is presented as

(36) $$G(k)={\left[\begin{array}{cccc} \sum_{t=1}^{l_{k}}a_{kt}s_{kt}^{1} & \sum_{t=1}^{l_{k}}a_{kt}s_{kt}^{2} & \ldots & \sum_{t=1}^{l_{k}}a_{kt}s_{kt}^{M} \end{array}\right]}^{T}$$

where *k* = 1, 2, …, *K*_1_.

Using (36), each column of the pairing matrix is constructed in a same structure to 1-D DE model defined in (1). This indicates that the pole $s_{kt}$ and $a_{kt}$, which correspond to the *k*-*th* pole $p_{k}$ in (32), can be solved by using 1-D SSA [22] to each column of the pairing matrix G. For example, for the first column of the pairing matrix G corresponds to $p_{1}$, the parameter matrices (**C**, **A**, **B**) can be obtained by using 1-D SSA in (4).

(37) $$G(1)={\left[\begin{array}{cccc} CB & CAB & \ldots & CA^{M-1}B \end{array}\right]}^{T}$$

The eigenvalue decomposition of the open-loop matrix A leads to.

(38) $$\left[\begin{array}{cccc} \tilde{y}(1) & \tilde{y}(2) & \ldots & \tilde{y}(N) \end{array}\right]=\left[\begin{array}{cccc} CB & CAB & \ldots & CA^{N-1}B \end{array}\right]$$

The matching pole $s_{1t}$ and the corresponding amplitude $a_{1t}$ are computed as.

(39) $$\left[s_{11},s_{12},\ldots ,s_{1l_{1}}\right]=\left[\begin{array}{cccc} \Lambda_{1}\left(1,1\right) & \Lambda_{1}\left(2,2\right) & \ldots & \Lambda_{1}\left(l_{1},l_{1}\right) \end{array}\right]$$

(40) $$\left[a_{11},a_{12},\ldots ,a_{1l_{1}}\right]={\left(CM_{1}\right)}^{*}\cdot \left(M_{1}^{-1}B\right)$$

Thus, the pairing matrix G can be searched column by column until all poles $s_{kt}$ and the corresponding amplitudes $a_{kt}$ are estimated. No extra pairing scheme is required because the poles $\left[s_{k1},s_{k2},\ldots ,s_{kl_{k}}\right]$ and the amplitudes $\left[a_{k1},a_{k2},\ldots ,a_{kl_{k}}\right]$ have already been adaptively paired to the poles $p_{k}$ in (34). In addition, considering that the model order may be overestimated sometime, the searched pole pairs are usually checked again according to their amplitudes. Finally, locations and damping factors of all the scattering points can be obtained from (41) and (42).

(41) $$\left(r_{1k},r_{2k})=(\frac{\mathrm{Arg}\{s_{k}\}}{4\pi f_{c}\Delta \theta /c},\frac{\mathrm{Arg}\{p_{k}\}}{4\pi \Delta f/c}\right)$$

(42) $$\left(\beta_{1k},\beta_{2k})=(\frac{\ln(\left|s_{k}\right|)}{f_{c}\Delta \theta },\frac{ln(\left|p_{k}\right|)}{\Delta f}\right)$$

## 4. Results and Discussion

In this Section, three examples are presented to demonstrate the usefulness of the proposed procedure, i.e., the numerical signatures with 14 point scattering centers, the numerical signatures of a sphere tipped cone-cylinder-frustum combination model, the measured ISAR data for an aircraft model. The results obtained by 2-D ESPRIT are used for comparison since it is one of the very few techniques which have been used in real radar applications [17,32].

### 4.1. Numerical Signatures with Point Scattering Centers

**Figure 1:**
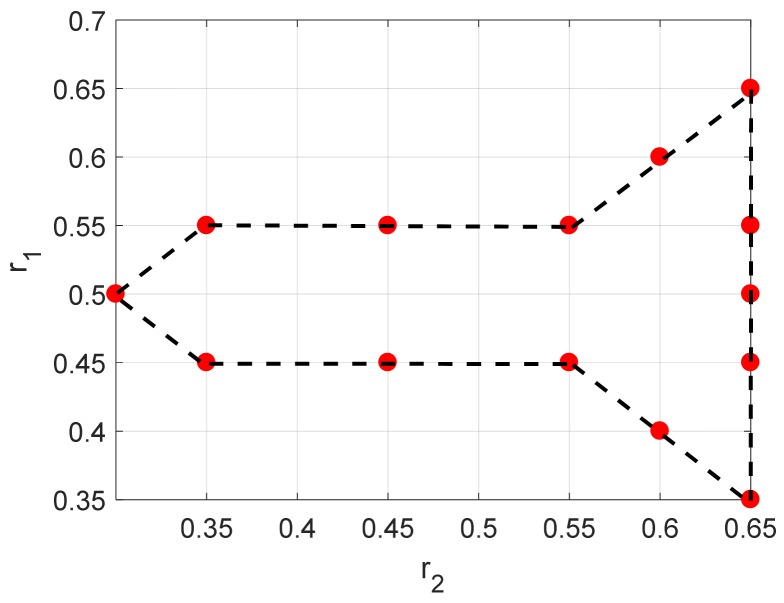
Spatial distribution of the simulated poles.

In this example, the noisy signatures composed of 14-point scattering centers are considered to be as follows:

(43) $$y(m,n)=\sum_{k=1}^{14}a_{k}\exp\left[\left(j2\pi r_{1k}/c+\beta_{1k}\right)f_{c}\theta_{n}\right]\exp\left[\left(j2\pi r_{2k}/c+\beta_{2k}\right)f_{m}\right]+w\left(m,n\right)$$

where (*M*, *N*) = (41, 41); $a_{k}$ is set to be 1; $f_{c}=12\;\mathrm{GHz}$ and Δf=150 MHz; $\theta_{0}=0$ rad and Δθ=0.0125 rad; The setting of coupled ranges are shown in Figure 1; All the damping factors including $\beta_{1k}$ and $\beta_{2k}$ are set as −0.05/$\left(f_{c}\Delta \theta \right)$ and −0.05/Δf except the far left scattering points ($\beta_{11}$, $\beta_{21}$) = (−0.02/$\left(f_{c}\Delta \theta \right)$, −0.02/Δf); w(m,n) denotes the additive white Gaussian noise.

**Figure 2:**
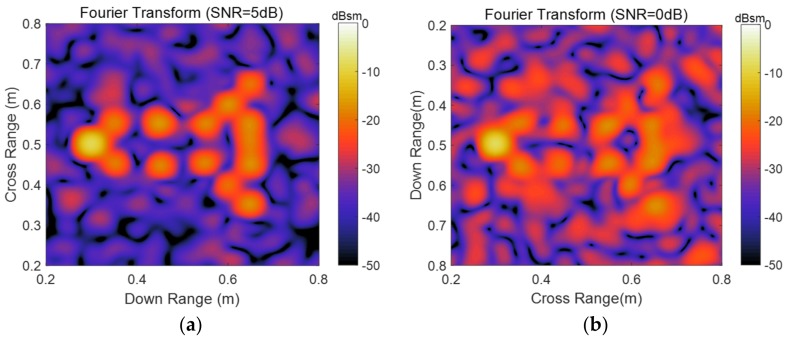
Simulated radar image with different SNR by Fourier transform. (**a**) Simulated radar image with SNR = 5 dB; (**b**) Simulated radar image with SNR = 0 dB.

**Figure 3:**
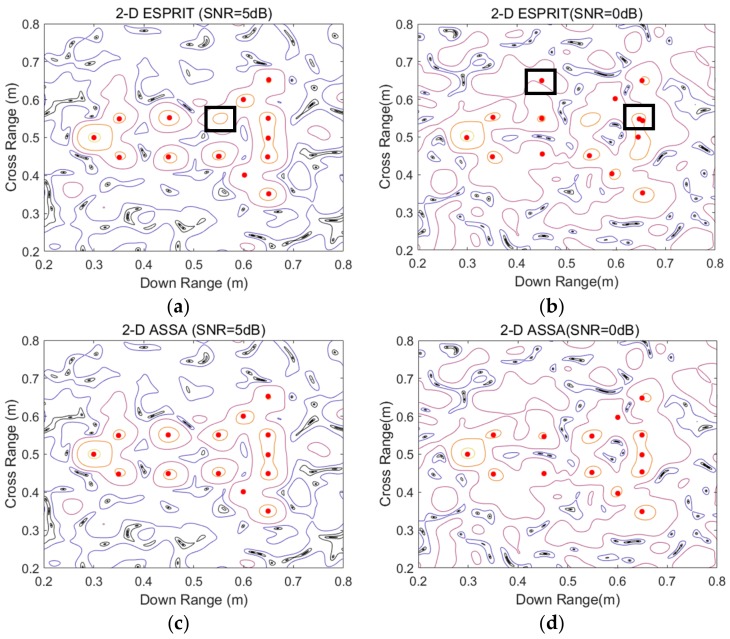
Estimation of the coupled ranges by 2-D ASSA and 2-D ESPRIT with different SNR. (**a**) Estimation by 2-D ESPRIT with SNR = 5 dB; (**b**) Estimation by 2-D ESPRIT with SNR = 0 dB; (**c**) Estimation by 2-D ASSA with SNR = 5 dB; (**d**) Estimation by 2-D ASSA with SNR = 0 dB.

As shown in Figure 1, these scattering points form a missile-like shape in Cartesian coordinates. It contains 13 pole pairs which have repeated poles in either the down-range or aspect dimensions. The noisy signatures in space domain by Fourier transform-based imaging algorithm (add Taylor window) are displayed in Figure 2a,b. As can be seen, the positions and decay rates of these scattering points are consistent with the corresponding ranges and damping factors. To sparse representation of these noisy signatures, the parameter for 2-D ASSA is chosen to be *L* = 20; the parameters for 2-D ESPRIT are set as: (*P*, *Q*) = (20, 20), β=0.8, which are suggested in [17]. For each signal to noise ratio (SNR), the number of Monte Carlo simulation is 200. A series of range estimation results in different SNRs are presented in Figure 3. Please note that the model order in 2-D ESPRIT is pre-specified because the singular values-based criterion [28,29] cannot be used when there are repeated poles in either the down-range or aspect dimensions. Because of different pairing strategies, *K*_1_ and *K* in 2-D ASSA could be estimated by the eigenvalue sequences transform algorithm (Appendix A) when SNR > 10 dB. However, the numbers *K*_1_ and *K* in 2-D ASSA should be pre-specified or use the other criterion [30] when the noise level is higher (SNR ≤ 10 dB).

As can be seen in Figure 3a,b, the positions of scattering points estimated by 2-D ESPRIT are generally according to the preset poles in Figure 1, except for some missing or incorrect points (shown by the black rectangular). The possible reason of this problem is that the pairing procedure in 2-D ESPRIT may provide incorrect pole pairs when there are repeated poles in either the down-range or aspect dimension, i.e., six pole pairs $\left(s_{1},p_{1}\right)$, $\left(s_{1},p_{2}\right)$, $\left(s_{1},p_{3}\right)$, $\left(s_{2},p_{1}\right)$, $\left(s_{2},p_{2}\right)$, $\left(s_{2},p_{3}\right)$. In contrast, the results estimated by 2-D ASSA showed higher accuracy than 2-D ESPRIT for different pairing strategies.

**Figure 4:**
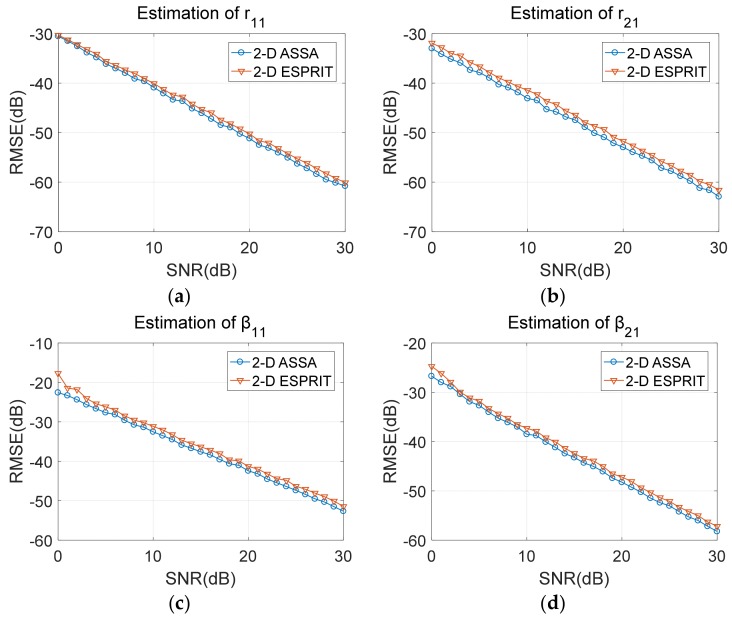
Estimation accuracy of *r*_11_, *r*_21_, *β*_11_ and *β*_21_ with different SNR. (**a**) Estimation of *r*_11_ with different SNR; (**b**) Estimation of *r*_21_ with different SNR; (**c**) Estimation of *β*_11_ with different SNR; (**d**) Estimation of *β*_21_ with different SNR.

The estimation accuracy of cross/down ranges and damping factors are displayed in Figure 4. Root mean square error (RMSE) is used as the evaluating indicator which is defined in (44). As we can see, estimation accuracy of these two algorithms are basically at the same level although the size of Hankel matrix used by 2-D ASSA is smaller than the block-Hankel matrix defined by 2-D ESPRIT.

(44) $$\delta_{RMSE}=10\log_{10}\sqrt{\frac{1}{M_{c}}\sum_{t=1}^{M_{c}}{\left[X_{\mathrm{est}}-X_{\mathrm{real}}\right]}^{2}}$$

where $M_{c}$ = 2000 denotes the number of Monte Carlo runs, $X_{\mathrm{est}}$ and $X_{\mathrm{real}}$ are the esimated and real parameters.

**Figure 5:**
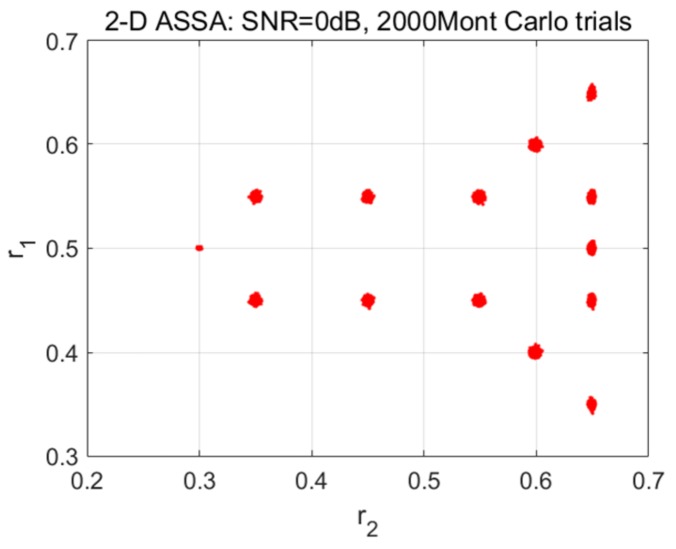
Estimation of the coupled ranges by 2-D ASSA with 2000 Monte Carlo runs (SNR = 0 dB).

Figure 5 also presents the statistic result with 2000 Monte Carlo runs (SNR = 0 dB). The result demonstrates that the multiple-range search strategy used by 2-D ASSA is robust in pole-pairing for low SNR.

### 4.2. Numerical Signatures of Computer-Aided Design (CAD) Model

**Figure 6:**
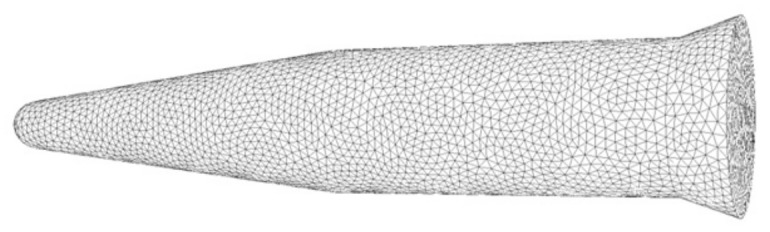
CAD model of the sphere tipped cone-cylinder-frustum combination.

**Figure 7:**
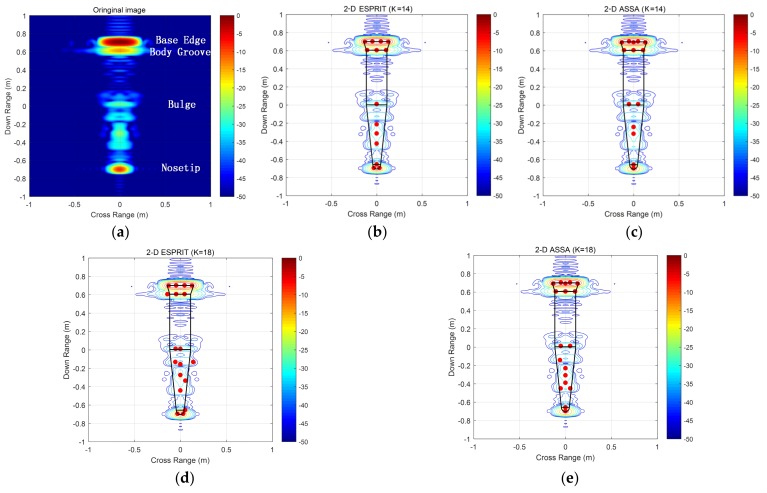
2-D radar image of the CAD model and location estimation of main scattering points result by 2-D ESPRIT and 2-D ASSA. (**a**) 2-D radar image in aspect angle from −5 to 5 deg; (**b**) Pole estimation by 2-D ESPRIT(*K* = 14); (**c**) Pole Estimation by 2-D ASSA(*K* = 14); (**d**) Pole estimation by 2-D ESPRIT(*K* = 18); (**e**) Pole Estimation by 2-D ASSA(*K* = 18).

The numerical signatures are obtained using method of moment (MOM), where the target is from a computer-aided design (CAD) model of a sphere tipped cone-cylinder-frustum combination, (shown in Figure 6). The data was calculated from 8–12 GHz in 10 MHz frequency step size, view angle ranging from −5 to 5 deg with an increment of 0.25 deg. Figure 7a presents 2-D radar image processed using 2-D FFT. As it can be seen, strong scattering points can be observed at differential discontinuities, such as the base-edge, the body groove, and nosetip. To sparsely represent the numerical data, the parameters of this example are set as follows. For 2-D ASSA, *L* is set as *N*2. For 2-D ESPRIT, (*P*, *Q*) = (*M*2, *N*2), where *M*2 = *ceil*(*M*/2) denotes the smallest integer less than or equal to *M*/2, and *β* = 0.8.

**Figure 8:**
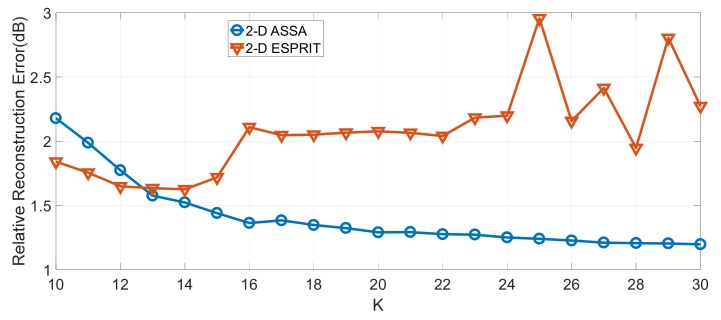
Relative reconstruction error of the numerical signatures of CAD model with different numbers of scattering centers.

Location estimation of key scattering points for these two algorithms are shown in Figure 7b–e. As can be seen, when the number of scattering centers is set to be 14, all key scattering points are accurately estimated by these two algorithms except for some minor differences. However, when *K* is set to be 18, 2-D ESPRIT encountered the pole-pairing problem and could not provide the right estimation. Relative reconstruction error (RRE) $\delta_{RRE}$ (defined in (45)) of these two algorithms are shown in Figure 8. We can see that the RRE of 2-D ASSA has been falling when *K* increased from 10 to 30, whereas the RRE of 2-D ESPEIT stopped falling after *K* = 14. The result shows that 2-D ASSA tends to be more robust in pole-pairing for different numbers of scattering centers.

(45) $$\delta_{RRE}=\frac{1}{MN}\sum_{m=1}^{M}\sum_{n=1}^{N}\left|20\log_{10}\left[Y_{recon}(m,n)/Y_{real}(m,n)\right]\right|$$

where **Y***_recon_* denotes the signatures reconstructed by the estimated parameters. **Y***_real_* is the original signatures.

**Figure 9:**
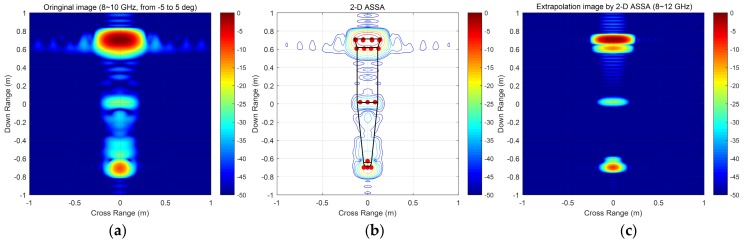
Sub-band data image and extrapolated data image by 2-D ASSA. (**a**) Sub-band data image by 2-D FFT (8–10 GHz); (**b**) Scattering points estimated by 2-D ASSA; (**c**) Full-band data image extrapolated by 2-D ASSA (8–12 GHz).

Figure 9a displays the sub-band data image (8–10 GHz, from −5 to 5 deg) which was extracted from the full-band data. Compared to the full-band data image in Figure 7a, scattering centers of the base-edge and the body groove cannot be distinguished when the signal bandwidth is only 2 GHz. Figure 9b presents the scattering points estimated by 2-D ASSA and Figure 9c shows the full-band data image extrapolated by these extracted pole pairs. As can be seen, scattering centers of the base-edge and the body groove are distinguished from the extracted scattering points. The full-band data image extrapolated by 2-D ASSA is in keeping with the main scattering centers distributed in the original full-band data image (Figure 7a). It is clear from these figures that 2-D ASSA results in higher-resolution images than traditional 2-D FFT imagery.

### 4.3. Measured ISAR Signatures

**Figure 10:**
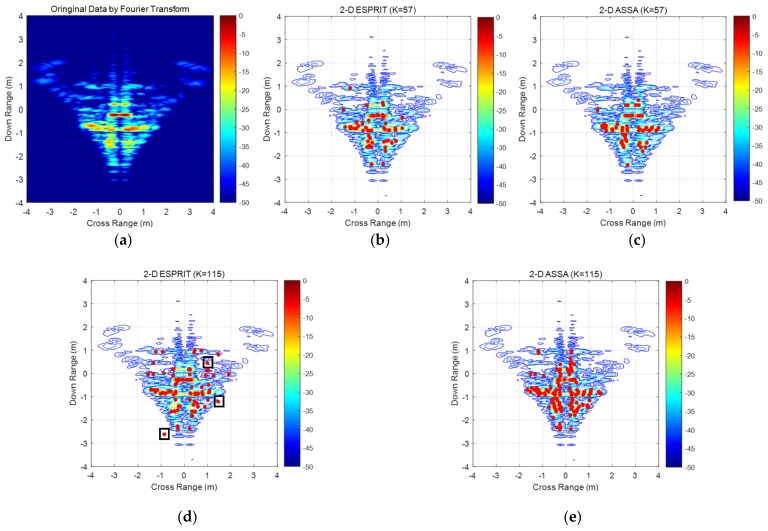
Pole-estimation result of the measured data with different *K*. (**a**) Measured ISAR image of the aircraft model by using 2-D FFT (**b**) Pole estimation by 2-D ESPRIT (*K* = 57); (**c**) Pole estimation by 2-D ASSA (*K* = 57); (**d**) Pole estimation by 2-D ESPRIT(*K* = 115); (**e**) Pole estimation by 2-D ASSA (*K* = 115).

In the third example, the measured ISAR signatures are acquired in an indoor test range where a mocked aircraft model is used. The data was collected using S-band radar with center frequency 3 GHz and 1.5 GHz bandwidth. The range of aspect angle is from −4° to 4° with an interval of 0.1°. Figure 10a shows the ISAR image of the aircraft model generated by 2-D FFT. As it can be seen, the scattering distribution of this model is more complex than the previous model, i.e., many scattering points are densely distributed in the fuselage. The estimation parameter for 2-D ASSA is set to be *L* = N2; For 2-D ESPRIT, (*P*, *Q*) = $\left(M2,N2\right)$ and *β* = 0.8.

Location estimation of main scattering centers for measured data are shown in Figure 10b–e. As we can see, both 2-D ESPRIT and 2-D ASSA could estimate the right positions of strong scattering centers. The scattering points estimated by 2-D ASSA are more densely distributed around the strong scattering centers than 2-D ESPRIT’s for different pole-pairing strategies. A few relatively weak scattering points, i.e., the red point in rectangle box, are wrongly estimated by 2-D ESPRIT when the number of scattering points is set to be 115, and more wrongly estimated pole pairs can be seen in *K* > 115 which are not displayed in Figure 10.

**Figure 11:**
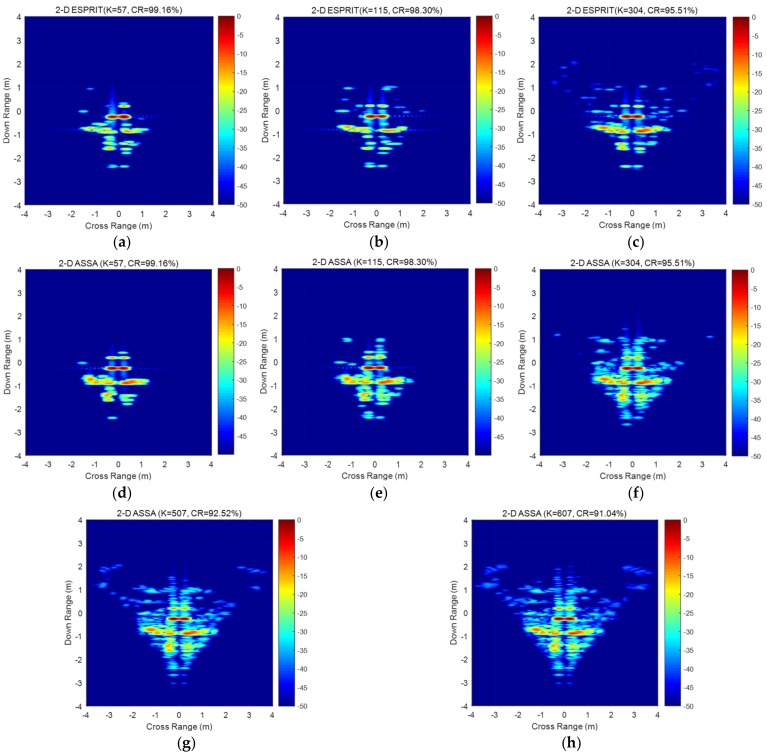
Reconstruction result of the measured data with different *K*. (**a**) Reconstruction result by 2-D ESPRIT(*K* = 57); (**b**) Reconstruction result by 2-D ESPRIT (*K* = 115); (**c**) Reconstruction result by 2-D ESPRIT (*K* = 304); (**d**) Reconstruction result by 2-D ASSA(*K* = 57);(**e**) Reconstruction result by 2-D ASSA(*K* = 115); (**f**) Reconstruction result by 2-D ASSA(*K* = 304); (**g**) Reconstruction result by 2-D ASSA(*K* = 507); (**h**) Reconstruction result by 2-D ASSA(*K* = 607).

Figure 11a–h display a set of reconstructed 2-D images by the estimated pole pairs. Please note that the number of scattering points *K* in 2-D ESPRIT should be equal to the model order whereas *K* in 2-D ASSA could be larger than the model order *K_1_* (the number of non-repeated poles in the down-range dimension) when there are one-to-multiple pairing poles. That is the reason *K* could be set to 607 which is already exceed the limitation of model order in [17]. As we can see from the figures, all those two algorithms can reconstruct those strong scattering centers of target which are in consistent with the estimated poles in Figure 10a–d. As displayed in Figure 11f–h, more and more relatively weak scatterin*g* centers have been reconstructed by 2-D ASSA with the increasing number of *K* and the reconstruction result with compression ratio of 91.04% is extremely similar to the original data in Figure 10a.

Evaluation of the reconstructed results by 2-D ASSA are listed in Table 2. Compression ratio (CR) $\varepsilon_{CR}$ and image similarity degree (ISD) are defined in (46) and (47). The RRE $\delta_{RRE}$ is used for evaluating the reconstructed result in frequency-domain, whereas the ISD $\gamma_{ISD}$ is for the result in image-domain. From the table, we can see that the numbers of scattering points in 2-D ASSA are negatively correlated with RRE (or ISD). In contrast, 2-D ESPRIT performs higher RRE than 2-D ASSA when the number of scattering centers remains the same. Please note that part of evaluation results by 2-D ESPRIT are abnormal because 2-D ESPRIT cannot do the right reconstruction when *K* > 307. The possible reason is that for 2-D ESPRIT, all amplitudes factors $\left[\begin{array}{cccc} a_{1} & a_{2} & \ldots & a_{K} \end{array}\right]$ (defined in (3)) are estimated together by using least square technique after all the pole pairs are confirmed. Thus, numerous incorrect pairing poles estimated by 2-D ESPRIT will lead to entirely wrong reconstruction. In comparison, 2-D ASSA performs better robustness for the measurement data.

(46) $$\varepsilon_{CR}=\frac{M\times N-n_{P}\times K}{M\times N}\times 100\%$$

(47) $$\gamma_{ISD}=\frac{\sum I_{recon}I_{real}}{\sqrt{\sum I_{recon}^{2}\sum I_{real}^{2}}}$$

where $n_{P}$ = 3 represents parameter number of each paired pole $\left(a_{k},s_{k},p_{k}\right)$, *K* is the number of scattering points, *M* × *N* denotes size of the original signatures. **I***_recon_*, **I***_real_* represent the reconstructed and original 2-D images-domain data.

Running time of the processed examples are listed in Table 3. All the results are carried out by Matlab (R2016b) with same hardware platform: Intel Core i7-6900k 3.2 GHz and 128 G memory. Please note that the running time of 2-D ESPRIT does not contain the estimation of model order. According to the table, 2-D ASSA performs higher efficiency than 2-D ESPRIT in processing the same data with the following differences.

(1) The size of the augmented Hankel matrix used by 2-D ASSA is approximate 1/(*M*/4) of the block-Hankel matrix used by 2-D ESPRIT when *L* = *Q* = *N*2 and *P* =*M*2. Moreover, the data size of the Hankel matrix could be further reduced by using the operation (22)–(24);

(2) No extra pairing scheme is required by using multiple-range search strategy which can finish the pairing process once the pole estimation of the aspect dimension is confirmed. Moreover, calculation of the block-Hankel matrix for the aspect dimension in 2-D ESPRIT is simplified as calculating multiple small Hankel matrices with size *M*2 × (*M* − *M*2 + 1).

## 5. Conclusions

In this paper, we have presented a two-dimensional augmented state–space approach for sparse representation of 2-D wideband radar signatures collected on man-made targets. To do this, a two-step procedure, i.e., an augmented state–space approach followed by multiple-range search strategy, is proposed to estimate the complex amplitudes and poles in down-range and aspect dimensions. In general, there are mainly two contributions provided in this paper. First, we develop a computationally efficient approach by adopting several time-saving operations, whereas the pole-estimation accuracy is still at the same level; second, the proposed approach can apparently alleviate the pole-pairing problem by using the multiple-range search strategy.

Numerical as well as measured ISAR data are processed to validate the proposed approach. Experimental results demonstrate that 2-D ASSA is robust and accurate in pole-paring for different SNRs, and is applicable for sparse representation of 2-D wideband radar signatures collected on man-made targets with low computational cost.

Future works are considered to be as follows. On the one hand, physical meanings of the extracted parameters of scattering centers by 2-D ASSA might be further studied to the possible applications in automatic target recognition (ATR). On the other hand, the extension of the proposed approach to 3-D radar signatures obtained from different azimuth and elevation angles would also be an interesting study.

## Figures and Tables

**Table 1:** Synopsis of the 2-D subspace-based algorithms.

Technique	Rationale	Pole ^(1)^ Pairing Scheme
2-D MUSIC	Signal-noise subspace decomposition	No need for pole-pairing
MEMP	Enhanced matrix decomposition	Maximizing a certain criterion
2-D TLS Prony	TLS-based Prony model	Minimizing a certain distance
ACMP	Signal subspace decomposition	Rank-restoration scheme
2-D ESPRIT	Shift-invariance structure of the signal subspace	Joint diagonalization scheme
2-D system realization	State–space model	Algebraic method

^(1)^. Pole denotes a transfer function of the scattering center collected on target. Usually, the scattering centers are characterized via pairing the complex amplitudes and the poles in down-range and aspect dimensions.

**Table 2:** Evaluation of the reconstruction result of the measured data by 2-D ESPRIT and 2-D ASSA.

	2-D ASSA		2-D ESPRIT	
Number of Scattering	$\delta_{RRE}(\mathrm{dB})$	$\gamma_{ISD}$	$\delta_{RRE}(\mathrm{dB})$	$\gamma_{ISD}$
*K* = 57 ($\varepsilon_{CR}$ = 99.16%)	3.26	0.90	3.93	0.88
*K* = 115 ($\varepsilon_{CR}$ = 98.30%)	2.60	0.92	3.20	0.90
*K* = 156 ($\varepsilon_{CR}$ = 97.70%)	2.53	0.92	3.11	0.90
*K* = 207 ($\varepsilon_{CR}$ = 96.95%)	2.47	0.93	2.96	0.91
*K* = 256 ($\varepsilon_{CR}$ = 96.22%)	2.27	0.94	2.94	0.91
*K* = 304 ($\varepsilon_{CR}$ = 95.51%)	2.07	0.94	2.90	0.91
*K* = 407 ($\varepsilon_{CR}$ = 93.99%)	1.76	0.95	41.28	0.26
*K* = 507 ($\varepsilon_{CR}$ = 92.52%)	1.60	0.96	68.69	0.12
*K* = 607 ($\varepsilon_{CR}$ = 91.04%)	1.51	0.96	43.57	0.24

**Table 3:** Running time of the numerical radar signatures and the measured ISAR data by 2-D ESPRIT and 2-D ASSA.

	Method	*K*	$\mathrm{Hankel}\;\mathrm{Matrix}\;(p_{k})$	$\mathrm{Hankel}\;\mathrm{Matrix}\;(s_{k})$	Running Time
Numerical data (41 × 401)	2-D ESPRIT	14	4000 × 4444	4000 × 4444	34.81 s
	2-D ASSA	14	8282 × 200	*K_1_* × 20 × 22	0.94 s
Measured ISAR data (81 × 251)	2-D ESPRIT	57	5000 × 5334	5000 × 5334	53.27 s
		115	5000 × 5334	5000 × 5334	94.18 s
	2-D ASSA	57	10287 × 125	*K_1_* × 40 × 42	1.04 s
		115	10287 × 125	*K_1_* × 40 × 42	1.19 s

## Appendix A

In the appendix, we provide an eigenvalue sequences transform algorithm to estimate the model order *K*_1_. Details are as follows.

Suppose that the size of the input matrix **H** is m×n(m≥n), do the SVD of the matrix $H^{*}H$.

(A1) $$H^{*}H=V{ℜ}^{2}V^{*}$$

where V is unitary matrix; ${ℜ}^{2}$ is the diagonal matrix.

$\Lambda =\left[\lambda_{1},\lambda_{2},,\ldots \lambda_{n}\right]$ is the diagonal vector of ℜ. It can be normalized as:

(A2) $$w_{i}=\frac{\lambda_{i}-\min(\Lambda )}{\max(\Lambda )-\min(\Lambda )}$$

where *i* = 1, 2, …, *n*; max(Λ) and min(Λ) denote the maximum and minimum elements of Λ.

The coordinate form of the normalized eigenvalue vector is:

(A3) $$\left[Q_{1},Q_{2},\ldots ,Q_{n}\right]=\left[\left(1,w_{1}\right),\left(2,w_{2}\right),\ldots ,\left(n,w_{n}\right)\right]$$

where $Q_{n}$ denotes the *n*-th points.

Based on information theory and principal factor analysis [28], the separable signals and noises in **H** often have the following hypothesis.

(A4) $$\begin{array}{c} w_{1}>\ldots >w_{K_{1}}>w_{K_{1}+1}\ldots >w_{n}\\ \mathrm{signal}\;\mathrm{space}|\mathrm{noise}\mathrm{space} \end{array}$$

(A5) $$w_{K_{1}}-w_{K_{1}+1}>w_{K_{1}+1}-w_{K_{1}+2}\approx \ldots \approx w_{n-1}-w_{n}$$

where *K*_1_ is the number of separable signals which also denotes the model order.

From (A5), it can be deduced that the point $Q_{K_{1}}$ belonging to $\left[Q_{1},Q_{2},\ldots ,Q_{n}\right]$ has the longest distance to line $Q_{1}D$ which has the following condition.

(A6) $$k\left(\vec{Q_{i}Q_{K_{1}+1}}\right)>k\left(\vec{Q_{1}D}\right)>k\left(\vec{Q_{K_{1}+1}Q_{n}}\right)$$

where i = 1, 2, …, *K*_1_, $k\left(\vec{Q_{i}Q_{K_{1}+1}}\right)$ denotes the slope of line $\vec{Q_{i}Q_{K_{1}+1}}$, *D* is a point which satisfies the slope condition (A6).

Here, an approximate calculation is used to estimate the slop of $\vec{Q_{1}D}$.

(A7) $$k\left(\vec{Q_{1}D}\right)\approx k\left(\vec{Q_{p-1}Q_{n}}\right)$$

(A8) $$g\left(p\right)=\max\left[g\left(1\right),g\left(2\right),\ldots ,.g\left(n\right)\right]$$

(A9) $$g\left(i\right)=\frac{\left|k\left(\vec{Q_{1}Q_{n}}\right)\left(i-1\right)-\left(w_{i}-w_{1}\right)\right|}{\sqrt{k{\left(\vec{Q_{1}Q_{n}}\right)}^{2}+1}}$$

where $Q_{p}$ represents the point in $\left[Q_{1},Q_{2},\ldots ,Q_{n}\right]$ which has the longest distance to line $Q_{1}Q_{n}$; $g\left(i\right)$ denotes the distance from point $Q_{i}$ to $\vec{Q_{1}Q_{n}}$; *i* = 1, 2, …, *n*.

Thus, $Q_{K_{1}}$ can be confirmed by searching the longest distance from point $Q_{i}$ to line $\vec{Q_{1}D}$.

(A10) $$g^{\prime }\left(i\right)=\frac{\left|k\left(\vec{Q_{1}D}\right)\left(i-1\right)-\left(w_{i}-w_{1}\right)\right|}{\sqrt{k{\left(\vec{Q_{1}D}\right)}^{2}+1}}$$

(A11) $$g^{\prime }\left(K_{1}+1\right)=\max\left[g^{\prime }(1),g^{\prime }(2),\ldots ,.g^{\prime }(n)\right]$$

where *i* = 1, 2, …, *n*; $g^{\prime }\left(i\right)$ denotes the distance from point $Q_{i}$ to $\vec{Q_{1}D}$.

## Appendix B

From the observability matrix Ω given by (20), controllability matrix Γ given by (21) and the matrices $\Omega_{rf}$, $\Omega_{rl}$, $\Gamma_{cf}$,$\Gamma_{cl}$ in (27),(28),(29) and (30), it is not difficult to deduce that the augmented open-loop matrix **P** satisfies the following matrix equations.

(A12) $$\Omega_{rf}P=\Omega_{rl}$$

(A13) $$P\Gamma_{cf}=\Gamma_{cl}$$

Then the augmented open-loop matrix **P** can be obtained by least squares.

(A14) $$P={\left(\Omega_{rf}^{*}\Omega_{rf}\right)}^{-1}\Omega_{rf}^{*}\Omega_{rl}$$

or

(A15) $$P=\Gamma_{cl}\Gamma_{cf}^{*}{\left(\Gamma_{cf}\Gamma_{cf}^{*}\right)}^{-1}$$

Moreover, here are two ways to compute the corresponding constant matrices **S** and **P**. The first way is for matrix **P** computed by (A14), the corresponding matrix **S** is defined as the first *M* rows of the observability matrix Ω.

(A16) $$S=\left[\begin{array}{ccc} \Omega \left(1,1\right) & \cdots & \Omega \left(1,K_{1}\right)\\ \vdots & \vdots & \vdots \\ \Omega \left(M,1\right) & \cdots & \Omega \left(M,K_{1}\right) \end{array}\right]$$

Using (A14) and (A16), we calculate the matrix $\tilde{\Omega }$

(A17) $$\tilde{\Omega }=\left[\begin{array}{cccc} S & SP & \ldots & SP^{N-1} \end{array}\right]$$

From (15) and (A17), the original 2-D signatures Y can be written as

(A18) $$Y=\tilde{\Omega }D$$

By using least square again, the matrix **D** is

(A19) $$D={\left({\tilde{\Omega }}^{*}\tilde{\Omega }\right)}^{-1}{\tilde{\Omega }}^{*}Y$$

The second way is for matrix **P** computed by (A15), the corresponding matrix **D** is defined as

(A20) $$D=\left[\begin{array}{c} \Gamma \left(1,1\right)\\ \vdots \\ \Gamma \left(K_{1},1\right) \end{array}\right]$$

Using (A15) and (A20), we calculate the matrix $\tilde{\Gamma }$

(A21) $$\tilde{\Gamma }=\left[\begin{array}{cccc} D & PD & \ldots & P^{N-1}D \end{array}\right]$$

Similar to (A18), the original 2-D signatures **Y** can also be rewritten as

(A22) $$Y=S\tilde{\Gamma }$$

By using least square, the matrix **S** is

(A23) $$S=Y{\tilde{\Gamma }}^{*}{\left(\tilde{\Gamma }{\tilde{\Gamma }}^{*}\right)}^{-1}$$

In this appendix, we provide two ways to estimate the parameter matrices (**S**, **P**, **D**). For consideration of the robustness to noise, (A23), (A14) (or (A15)) and (A19) are preferred. It is also worth mentioning that 2-D ASSA has two ways, which include the matrix form (**S**, **P**, **D**) and the pole form $\left(a_{k},s_{k},p_{k}\right)$, to reconstruct the original signatures **Y**. The matrix form could provide more accurate reconstruction result than the pole form but with much more parameters.
